# Cerebellar contribution to the regulation of defensive states

**DOI:** 10.3389/fnsys.2023.1160083

**Published:** 2023-03-31

**Authors:** Gabriela Neubert da Silva, Nina Seiffert, Philip Tovote

**Affiliations:** ^1^Defense Circuits Lab, Institute of Clinical Neurobiology, University Hospital Würzburg, Würzburg, Germany; ^2^Center for Mental Health, University Hospital Würzburg, Würzburg, Germany

**Keywords:** cerebellum, PAG, amygdala, prefrontal cortex, heart rate, fear, defensive states, prediction error

## Abstract

Despite fine tuning voluntary movement as the most prominently studied function of the cerebellum, early human studies suggested cerebellar involvement emotion regulation. Since, the cerebellum has been associated with various mood and anxiety-related conditions. Research in animals provided evidence for cerebellar contributions to fear memory formation and extinction. Fear and anxiety can broadly be referred to as defensive states triggered by threat and characterized by multimodal adaptations such as behavioral and cardiac responses integrated into an intricately orchestrated defense reaction. This is mediated by an evolutionary conserved, highly interconnected network of defense-related structures with functional connections to the cerebellum. Projections from the deep cerebellar nucleus interpositus to the central amygdala interfere with retention of fear memory. Several studies uncovered tight functional connections between cerebellar deep nuclei and pyramis and the midbrain periaqueductal grey. Specifically, the fastigial nucleus sends direct projections to the ventrolateral PAG to mediate fear-evoked innate and learned freezing behavior. The cerebellum also regulates cardiovascular responses such as blood pressure and heart rate-effects dependent on connections with medullary cardiac regulatory structures. Because of the integrated, multimodal nature of defensive states, their adaptive regulation has to be highly dynamic to enable responding to a moving threatening stimulus. In this, predicting threat occurrence are crucial functions of calculating adequate responses. Based on its role in prediction error generation, its connectivity to limbic regions, and previous results on a role in fear learning, this review presents the cerebellum as a regulator of integrated cardio-behavioral defensive states.

## 1. Introduction

In higher organisms, complex states of the nervous system have evolved as adaptive responses to fluctuating threats, in order to increase chances of survival (LeDoux, 2012; Koutsikou et al., 2014). These evolutionary conserved defensive states can turn maladaptive in humans in the form of fear and anxiety-related disorders (American Psychiatric Association, 2013). The rising prevalence of these disorders calls for a better understanding of these disorders through rigorous mechanistically oriented research (Kessler et al., 2005). A defensive state depends on multimodal, highly dynamic processes between the brain and the body, which are reflected by adaptations of behavioral, cardiovascular, and endocrine changes (LeDoux, 2000). Whereas the central nervous system acts as a command center for defensive responses, it also receives interoceptive bodily information, which depend on defensive state dynamics (LeDoux, 2000). Thus, part of the complexity of defensive states is given by the need for processing and integration of different exteroceptive and interoceptive inputs to form a coherent model of the threat situation, as a prerequisite for adaptive threat coping strategies.

One of the core tasks that is classically assigned to the cerebellum is sensory-motor integration, which is thought to be achieved by computing (sensory) prediction errors (Wolpert et al., 1998). More specifically, the cerebellum makes use of predictive models, which generate and compare expected outcomes with the input of the actual state of the environment (Ito, 1970, 1972). Any mismatch triggers a prediction error, which in turn leads to the adaptation of the output. In addition to this fast “online prediction” mechanism for moment-to-moment adaptation, i.e., fine-tuning of motor patterns, the cerebellum seems to contribute prediction errors to drive long-term plasticity during associative learning.

This review will present recent evidence for a role of the cerebellum in regulating defensive states *via* prediction error mechanisms. It has been demonstrated that the cerebellum is involved in fear learning, which in turn has been mechanistically explained by models involving prediction errors (Sacchetti et al., 2004; Timmann et al., 2010; Popa and Ebner, 2019). It is furthermore known that the deep cerebellar nuclei (DCN), the three output structures of the cerebellum, are neuroanatomical connected to circuits which regulate defensive states (Koutsikou et al., 2014; Frontera et al., 2020; Vaaga et al., 2020; Lawrenson et al., 2022). However, it is unclear so far to which degree the cerebellum is involved in the regulation of sensory-motor integration specific to defensive states. Moreover, it still needs to be determined whether cerebellar contributions are involved in regulating innate defensive states in addition to their role in the regulation of learned defensive states.

## 2. The cerebellar mechanisms of temporal motor control and learning

It has been suggested that one of the central operating mechanisms of the brain is to generate predictions about the world in order to adapt future behavior accordingly (Keller and Mrsic-Flogel, 2018). For successful adaptation, the brain relies on the computation of prediction errors, which constitute the difference between the brain’s prediction of a variable and the actual input related to that variable. Traditionally, the cerebellum has been described as the key player in sensory-motor integration, being specifically responsible for motor control and motor learning (Keller and Mrsic-Flogel, 2018; Hull, 2020). The cerebellum is thought to achieve this by utilizing predictive models which generate and compare expected outcomes of motor behavior with the actual outcome (Ito, 1970, 1972). It is therefore also described as the place of acquisition and storage of internal/forward models of the motor system (Popa and Ebner, 2019).

The cerebellum uses internal models to calculate motor outputs according to sensory inputs (inverse model) and to anticipate future sensory inputs based on previous motor outputs (forward model) (Wolpert et al., 1998). However, this motor planning can result in errors. The cerebellum tries to reduce the chance of an error occurring by different mechanisms: feedforward control, feedback control, and sensory prediction. The first, feedforward control, is generated without taking into account any output and consequently does not allow the computation of an “online” error. This can be advantageous when a fast motor response is needed. Furthermore, feedforward control is responsible for initiating motor actions. Feedback control, on the other hand, generates corrections in case an error occurs. The last mechanism, sensory prediction, combines prediction and sensory feedback to estimate forward outputs, thereby attempting to provide an accurate movement. When an error occurs, i.e., the predicted output does not match the initial motor command, an online corrective motor output may be generated while the movement progresses (Wolpert et al., 1998; Blakemore et al., 1999).

These cerebellar mechanisms have most extensively been investigated in the context of motor control and motor learning. The cerebellum plays a role in both implicit information processing, i.e., automatic skill/motor adaptation, as well as in explicit information processing, i.e., learning under conscious control. More specifically, implicit learning is described as a process that is driven by sensory prediction errors thereby being relatively independent of the context, but a slow and steady process. Task performance errors on the other hand are thought to drive explicit learning, which is described as faster than implicit learning, exploratory, and underlies conscious control (Popa and Ebner, 2019). This type of prediction error is mostly used in learning to update already existing or create new models about motor behaviors. The cerebellum is thought to realize this by a supervised learning rule, in which the confirmation of whether or not the outcome matches the expectations is conveyed *via* teaching signals. More precisely, it is thought that climbing fiber (CF) input to the cerebellar cortex informs the cerebellum about occurring movement errors. The correction of future movements is thought to be implemented by large dendritic calcium spikes (complex spikes), which are generated by the aforementioned error signals in the Purkinje cells (PC) (Marr, 1969; Albus, 1971; Hull, 2020). However, recent research has uncovered that the classical cerebellar supervised learning models are not sufficient to explain certain cell activity, more precisely the CF-driven complex spikes in PCs (Kitazawa et al., 1998; Streng et al., 2018; Hull, 2020). Instead, the temporal difference models of reinforcement learning seem to be able to describe this specific cell activity more accurately. Here, the teaching signals are scalar, thereby varying depending on current expectations. Furthermore, the teaching signals possess properties that allow to not only change what but also how a system learns. Modulation of the teaching signals occurs by experience to represent higher-order reinforcing stimuli (Sutton and Barto, 1998; Brinke et al., 2015; Hull, 2020). These different properties of the teaching signals enable learning *via* trial-and-error exploration without needing any pre-existing knowledge about a correct outcome (Hull, 2020). Beyond the well- established role of cerebellar prediction mechanism in sensory-motor integration and motor learning, recent studies have found evidence that supports the notion of cerebellar contribution to reward-based reinforcement learning (Heffley et al., 2018; Heffley and Hull, 2019; Kostadinov et al., 2019; Larry et al., 2019). Yet, the cerebellar role in learning was largely evaluated in paradigms using aversive conditions and cues.

## 3. Cerebellar regulation of learned behavioral responses to threat

### 3.1. Intra-cerebellar microcircuits

A learning paradigm that induces associations of negative valence is eyeblink conditioning which typically consists of a tone (CS) paired with an air puff or a periorbital shock (US). In contrast to fear conditioning, acquisition of the eyeblink/eyelid response takes hundreds of training trials. Studies in rabbits in the 1980’s revealed that the cerebellum is critically involved in eyeblink conditioning [for review, see Medina et al. (2002)]. Bilateral lesions within the IN extending to the DN prevented the acquisition and abolished the expression of the eyelid conditioned response (CR) (Lavond et al., 1984). In addition, more precise lesion experiments demonstrated that the anterior IN (aIN) is essential for CR retention (Lavond et al., 1985; Clark et al., 1992; Clark and Lavond, 1993; Nordholm et al., 1993). Studies addressing intra-cerebellar circuitry involved in eyeblink conditioning found that US input to the aIN is conveyed *via* CF system of the inferior olive (Mauk et al., 1986; Sears and Steinmetz, 1991), while the CS information is transmitted *via* the mossy fiber system of the pontine nucleus (Sears and Steinmetz, 1991; Clark and Lavond, 1993; Krupa et al., 1993; Krupa and Thompson, 1995; Rasmussen et al., 2008). Taken together, early results obtained from rabbits and later complementary findings in rats (Brinke et al., 2017) provide conclusive evidence for a role of the aIN as a crucial site for acquisition, storage, expression, and extinction of eyeblink conditioning memory (Brinke et al., 2017).

The functional involvement of the fastigial nucleus (FN) in eyeblink conditioning was recently investigated in detail by using electrophysiological, tracing, chemo-, and optogenetical approaches (Wang et al., 2020). FN glutamatergic neurons and their upstream PCs within vermal lobules IV-VIII showed modulatory activity after the CS presentation, which was correlated with the CR amplitude on a trial-by-trial basis. It was furthermore demonstrated that the FN projects to the ventral medullary reticular nucleus. In well-conditioned animals, optogenetic suppression of this pathway impaired both CR probability and amplitude as well as UR amplitude. Interestingly, the FN-ventral medullary reticular nucleus pathway projects to the same 7N motoneurons (responsible for eyelid movements) as the IN-red nucleus pathway, which is known for being crucial for driving the CR in eyeblink conditioning. Therefore, it was hypothesized that the distinct FN and IN pathways need to be coactivated to synergically trigger adequate commands in the 7N motor neurons (Wang et al., 2020).

Eyeblink conditioning is also dependent on the cerebellar cortex, which exerts a tonic inhibition on the DCN. Optogenetic suppression of PCs in lobules HV and HVI was sufficient to drive eyeblink behavior in mice (Heiney et al., 2014). Accordingly, optogenetic stimulation of mice PCs during the CS-US pairing interval also abolished the CR (Brinke et al., 2017). These results are in line with earlier studies in rabbits, which have shown that PCs in lobe HVI presented an activity reduction, i.e., long-term depression (LTD), while aIN cells boosted their activity, i.e., long-term potentiation (LTP) during the paradigm (Kim and Thompson, 1997; Ohyama et al., 2006). Interestingly, a recent study in mice showed that the extracellular matrix surrounding the cerebellum modulates GABAergic transmission from PCs to the DCN (especially the IN), interfering with IN LTP and, therefore, with learning of eyeblink conditioning (Hirono et al., 2018).

Beyond the strengthening and weakening of intra-cerebellar synaptic connections, several studies have shown that the cerebellum may play a role in learned defensive responses by predicting and transmitting error signals to other brain regions, such as it occurs in motor control (Brinke et al., 2015; Ohmae and Medina, 2015). As such, these prediction errors function as teaching signals, important for triggering plasticity within a larger network (for review, see McNally et al., 2011). Climbing fiber inputs onto PCs were discovered to exhibit an increase in their firing rate in response to an unexpected US presentation, an inhibited firing rate when faced with an expected US presentation, and to fire during CS prediction in well-trained eyeblink conditioned mice (Ohmae and Medina, 2015). These findings indicate that the inferior olive encodes prediction error signals during associative tasks which are dependent on the cerebellum, thereby driving adaptive learning. Moreover, in another study which manipulated the IN-inferior olive pathway and recorded PCs receiving climbing fiber projections during eyeblink conditioning, stimulation of inhibitory IN- inferior olive projections prevented PC activity responses to the US in naïve animals, demonstrating a local feedback circuitry responsible for an online CR modulation. Stimulation of the same inhibitory IN-inferior olive pathway in well-conditioned mice during but not after CS-US pairing extinguished the triggering of behavioral and cellular CR similarly as in WT animals during the extinction phase (Kim et al., 2020). That is, even with the continued US presentation, the inhibition of the inferior olive by the IN was able to inhibit the CR. These results suggest there are online prediction error signals driven by the inferior olive, which may play a central part in modulating learned defensive responses.

The PC activity mediated by climbing fibers affects aIN activity during conditioning (as previously mentioned), possibly in combination with activation of the mossy fiber and climbing fiber collaterals. It was therefore suggested that climbing fiber input to the cerebellar cortex, together with direct input to the aIN, provides a modulation of aIN activity to fine-tune CR timing (Brinke et al., 2017). In fact, cerebellar cortical learning was studied using electrical stimulation of mossy fibers as the CS and stimulation of climbing fibers as the US with different interstimulus intervals in decerebrated ferrets. PCs belonging to the C3 zone (which send projections to the IN) presented either sequential CRs paused according to each interval or long-duration paused CRs that span both interstimulus intervals. Moreover, in the case of the paused CRs, the learning process of each CR was different: the first CR occurs earlier in time than the second CR. These results suggest that C3 zone PCs learned associations between two sequential stimuli presented with at least two distinct intervals, responding accordingly to each one. Interestingly, the second CR is not a simple repetition of the first one, but an independent response by itself (Jirenhed et al., 2017). Therefore, PCs can learn timed sequential response patterns, suggesting greater importance of internal mechanisms for cerebellar control and regulation of motor defensive behaviors, possibly through prediction error signals.

In contrast to other cerebellar neurons, the granule cells are usually silent at rest and are activated by mossy fibers inputs with a fast spike initiation, thereby ensuring almost instantaneous PC activation (D’Angelo et al., 1995).

Their modulation occurs *via* different excitatory and inhibitory neurotransmitter receptors operating in a double time band, which indicates the granule cells as a potential spike-time controller (Bareš et al., 2019). The acquisition of precise timing is suggested to first occur within the granular layer, providing the time precision to cerebellar computations.

In accordance with the animal studies, an fMRI study in humans showed activation of lobules Crus I and VI in both situations: under CS presentation and during an unexpected omission of the US in the acquisition phase of fear conditioning. During the extinction phase on the other hand, when US omission became expected, the cerebellar cortex was not recruited (Ernst et al., 2019).

In addition to its role in conditioning of the eyeblink reflex, many animal studies used classical auditory Pavlovian conditioning to address the cerebellum’s role in associative learning. Auditory fear conditioning is especially suited to address learned responses to acute threatening cues and contexts. It induces an associative learning process, through which the animal learns to express a CR in response to the presentation of a conditioned stimulus (CS) which is paired with an aversive, unconditioned stimulus (US) (Fendt and Fanselow, 1999; LeDoux, 2000; Maren, 2001). The paired presentation of CS and US is called the acquisition phase. Subsequently, consolidation processes result in long-term associative memory of the CS and conditioning context. In the retrieval phase, the animals will express the CR upon sole presentation of the CS, or when re- exposed to the conditioning context. Further repetitive presentation of the CS or context alone will trigger extinction learning, which results in reduction of the CR (Tovote et al., 2015). Forming associative threat memories is a fundamental process in which experience is used to optimize coping with threat, and many brain regions, most prominently the amygdala, the hippocampus, and the prefrontal cortex have been demonstrated to be essential for this type of learning.

More recently, the cerebellum has increasingly been recognized for its contributions to associative threat learning (Apps and Strata, 2015). In rats, pharmacological inactivation of the vermis and IN during the consolidation phase impaired threat memory (Sacchetti et al., 2002). Moreover, the vermis has also been demonstrated to be necessary for contextual threat memory consolidation (Sacchetti et al., 2002). Vermal LTP (in lobes V and VI) between parallel fiber (PF)-PC excitatory synapses was observed 10 min and 24 h after rats underwent fear conditioning. The LTP only occurred in rats who received paired CS-US presentation in fear conditioning, but not in those in which the CS-US presentation was unpaired (Sacchetti et al., 2004). Mice with a deficiency of the PF-PC synapses presented a decrease in freezing response when tested 10 min and 24 h after the acquisition phase (Sacchetti et al., 2004). Furthermore, induced LTP at the PF–PC excitatory synapses, but not LTD, was decreased 10 min and 24 h after conditioning in CS-US paired rats (Zhu et al., 2007). Using the same protocol, it was further demonstrated that fear conditioning also induces pre-synaptic LTP of GABAergic synapses from molecular layer interneurons onto the PCs (Scelfo et al., 2008). In contrast, a recent study that evaluated the role of inhibitory synapses in fear conditioning by using transgenic mice lacking the GABAA receptor γ2 subunit specifically in PCs found no significant differences between transgenic mice and controls (Marshall-Phelps et al., 2020). Taken together, these findings indicate that LTP in both excitatory and inhibitory synapses onto PCs is related to associative short- and long-term fear memory, with the balance between excitatory and inhibitory inputs to the PCs being a putative mechanism for spatiotemporal firing control.

The importance of PCs in fear conditioning was confirmed by the results of a recent study which investigated the effects of spinocerebellar ataxia, a neurodegenerative condition affecting the vermis and intermediate parts of the cerebellar cortical hemispheres, in cognition and fear memory. An impairment of the threat-induced freezing response during the acquisition phase and during the recall session on the next day was found in a transgenic mouse line in which the expression of pathological Ataxin 1, the gene triggering the onset of spinocerebellar ataxia, was restricted to cerebellar PCs. Strikingly, mice with unspecific expression of the mutant gene throughout the entire brain, showed no effects in freezing response during the acquisition, while freezing responses were even lower during the recall phase (Asher et al., 2020). Besides reinforcing the role of PCs in fear conditioning, these results also suggest that spinocerebellar ataxia can provoke fear memory deficits triggered by a combination of cerebellar and extra-cerebellar malfunctions. Mechanistically, a critical role of CF in controlling the induction of LTP and LTD at the PF–PC synapses was suggested (Coesmans et al., 2004).

### 3.2. Cerebellar (functional) connections with emotion-processing networks

To elucidate that cerebellar microcircuits are part of a larger brain network for threat learning (Han et al., 2021), transsynaptic tracers have proven powerful tools in addressing the neuroanatomical basis for cerebellar defensive networks. In order to trace a putative pathway involved in eyeblink conditioning, an attenuated pseudorabies virus (PRV) was injected into the rabbit orbicularis oculi (eyelid) muscle (Gonzalez-Joekes and Schreurs, 2012). The retrograde labeling first reached the facial nucleus, then the medullary nuclei, including the nucleus of the solitary tract (NTS), subsequently the aIN, FN, periaqueductal gray (PAG), and other motor nuclei, and lastly the rest of the IN, the DN and the cerebellar cortex (lobules I and HVI), the cuneate nucleus, the gracile nucleus, the deep mesencephalic nucleus, the retrorubral field, the superior colliculus and the substantia nigra (Gonzalez-Joekes and Schreurs, 2012).

Similar results were also found in rats (Morcuende et al., 2002), indicating that a network beyond cerebellar-olivary circuits is involved in eyeblink conditioning responses. Modern genetical approaches shed light not only on connectivity between brain regions, but allow for precise characterization of connections between individual circuit elements, i.e., localized neuron populations with known molecular identity.

To this end, a recent study has identified subtypes of glutamatergic FN neurons with defined molecular profiles, sizes, topographical organization (Fujita et al., 2020). Moreover, this study demonstrates distinct organization of FN neurons with respect to specific input from cerebellar lobules and vermis, thereby adding complexity, i.e., modularity, on the level of the DCN to the known pathways mediating cerebellar functions. The authors hypothesize that due to their innervation by fast spiking PCs, large size and output to specific brainstem centers, rostral FN neurons specialize in transmitting precisely timed information important for online control of somatomotor, autonomic and arousal outputs. Other, smaller FN neurons not innervated by fast spiking PCs are thought to mediate tonic modulation of autonomic functions and vigilance and arousal (Fujita et al., 2020).

#### 3.2.1. Midbrain periaqueductal gray

Building on tracing studies that demonstrated tight connections between the cerebellum and midbrain structures important for defensive behaviors, several studies specifically addressed DCN-PAG connectivity. Using anterograde, retrograde, and transsynaptic viral vector-mediated strategies to trace glutamatergic and GABAergic IN inputs and outputs, Judd et al. found that both neuron types project from the PAG to the IN. Furthermore, discrete projections from the NTS to the IN were seen (Judd et al., 2021), beyond other motor and sensory areas already known for projecting to the cerebellum. Interestingly, the IN was shown to send glutamatergic and GABAergic projections to the PAG as well (Judd et al., 2021). However, whether and how this bidirectional connectivity is involved in eyeblink conditioning is still unknown. Electrical stimulation of the ventrolateral PAG (vlPAG) in rats evoked field potentials in vermal lobule VIII, also known as the pyramis (Koutsikou et al., 2014).

Moreover, pyramis lesions induced by saporin conjugated with the labeling marker cholera toxin subunit B impaired both, conditioned freezing to an auditory CS in fear conditioning, as well as freezing in response to a cat-odor stimulus (Koutsikou et al., 2014). Further studies have been concerned with identifying the circuitry involved in these defensive behaviors, addressing cell type specificity and functionality. Glutamatergic FN neurons send projections to the vlPAG, targeting excitatory glutamatergic and inhibitory GABAergic as well as neuromodulatory dopaminergic neurons (Frontera et al., 2020; Vaaga et al., 2020). The activation of the glutamatergic FN-vlPAG pathway during the CS-US pairing in the acquisition phase, but not in the consolidation phase impaired CR retrieval in fear conditioning (Frontera et al., 2020). Strikingly, in well-conditioned mice, chemogenetics activation of the FN-vlPAG pathway during the entire first extinction session did not affect extinction, but its inhibition reduced memory extinction.

Temporally precise optical stimulation of this pathway at CS offset, however, enhanced fear extinction (Frontera et al., 2020). These results suggest that the FN-vlPAG pathway regulates learned defensive responses by modulating the CS-US association, thereby controlling memory formation. In addition, it plays a role in regulating fear extinction, with temporally precise activity required at the time of the negative prediction error. In line with these results, inactivation of the FN-vlPAG pathway during the acquisition phase reduced the occurrence of threat-induced ultrasonic vocalizations during the intertrial interval in the acquisition phase and slowed down the extinction rate in rats (Lawrenson et al., 2022). These behavioral disruptions seen in different phases of fear conditioning raise the possibility that the FN regulates different aspects of defensive behaviors. For a better understanding whether and how FN modulates vlPAG activity in fear conditioning, Lawrenson et al. performed single-unit recordings in rats (Lawrenson et al., 2022). FN neural activity resembled increased vlPAG activity upon an auditory CS onset and offset during the extinction phase of conditioning (but not during the entire CS). Chemogenetic inactivation of the FN during the consolidation phase resulted in an increase of vlPAG CS offset activity, but not onset activity, and a delay in the latency peak during the early extinction phase. Hence, vlPAG neuronal response patterns at CS onset and offset are likely generated by different neural pathways, with the temporally precise CS offset neural activity being influenced by FN. Additionally, vlPAG CS offset activity enhancement during extinction was accompanied by an increase in the duration of individual freezing bouts. These results support a role of the cerebellum in temporally precise modulation of PAG neuronal activity.

#### 3.2.2. Amygdala

The amygdala, with its lateral (LA), basolateral (BLA), and central nuclei (CEA) has long been known as a central player in innate and learned defensiveness, and aversive emotions such as fear and anxiety. Although there is no known monosynaptic connection between amygdala nuclei and the cerebellum, there is ample evidence for a functional relationship of the two structures. Importantly, the amygdala has been demonstrated to be involved in eyeblink conditioning and putative connectivity with the cerebellum has been suggested (Mintz and Wang-Ninio, 2001; Lee and Kim, 2004; Farley et al., 2016; Frontera et al., 2020). Lesions and pharmacological inactivation of the basolateral amygdala (BLA) before the training session of eyeblink conditioning have been shown to slow the acquisition of the eyeblink CR (Lee and Kim, 2004). In fact, based on both eyeblink and heart rate conditioning assays, some studies have suggested that, whereas the aIN is crucial for the expression of the CR, the amygdala modulates the acquisition of the CR, thereby facilitating its expression (Lavond et al., 1984; Kim and Thompson, 1997; Mintz and Wang-Ninio, 2001). Therefore, a putative connectivity from the amygdala to the cerebellum was thought to play a modulatory role during the learning process of defensive behaviors. However, further studies performed in rats have demonstrated that CEA activity seems to be important even after learning (Farley et al., 2016), thereby supporting a role for the CEA in sensory gating *via* the CS pathway (i.e., through the pontine nucleus) (Taub and Mintz, 2010). Thus, the short-term associative process within the CEA facilitates neuronal activity within the pontine nucleus in response to the CS, which in turn facilitates the cerebellar motor CR (Steinmetz et al., 2017), suggesting a pathway involved in learned defensive behaviors. Indeed, CEA neurotoxic lesions impaired freezing, delayed eyeblink CR, and reduced the number of cFos expression within the pontine nucleus of rats that underwent 4 sessions of delay eyeblink conditioning (Pochiro and Lindquist, 2016). However, another study has found an inhibition of CEA firing response to a periorbital shock in rats under electrical stimulation of the FN and IN, suggesting a cerebellar modulation of amygdala-dependent threat learning (Magal and Mintz, 2014).

These results are in line with a study by Wang et al., which suggests both, FN and IN, need to be coactivated in order to trigger an adequate CR (Wang et al., 2020). Moreover, cFos expression was diminished in the CEA, BLA, and LA of sham rats after 4 days of delayed eyeblink conditioning (Pochiro and Lindquist, 2016). Human fMRI studies confirmed these results obtained in animal models (Ernst et al., 2017). In healthy subjects performing eyeblink conditioning, the activity within the cerebellar cortex and DCN (IN and DN) was increased during the acquisition and reacquisition phases, but not during extinction, consistent with bidirectional learning (Pochiro and Lindquist, 2016; Ernst et al., 2017; Lindquist, 2020).

Additionally, previous studies investigated the putative connection between the BLA and the cerebellum (Lee and Kim, 2004). Specifically, BLA activity is required during the acquisition and early consolidation phase of fear conditioning for enabling PF-PC vermal LTP (Zhu et al., 2011). The authors hypothesized that this learning-induced vermal LTP enables the CS to activate PCs, thereby allowing the selection of a more appropriate response to the CS. In line, the BLA and the vermis are required for memory retention after fear memory consolidation. Inactivation of both during the recall session caused long-lasting amnesic effects, since rats did not recover the fear memory over time, not even after a reminder shock (Sacchetti et al., 2007). The advancement of neuroscientific methodologies recently allowed the demonstration of disynaptic connections between the DCN (FN and IN) and the BLA in mice, synapsing within the centromedial and parafascicular thalamic nuclei (Frontera et al., 2020; Jung et al., 2022). Due to the cerebellum’s role in prediction error generation (Popa and Ebner, 2019) and the emotional valence information encoded by BLA (Janak and Tye, 2015), this pathway may be involved in encoding the information about the prediction and valence of a US. Overall, bidirectional functional connectivity between the amygdala and the cerebellum and evidence for disynaptic anatomical connectivity *via* the intralaminar thalamus make a strong case for a role of specific cerebellar-amygdala circuits in regulation of adaptive defensive states.

#### 3.2.3. Prefrontal cortex

Presenting another important node in brain networks for defensive states, the medial prefrontal cortex (mPFC) has been implicated in a more complex type of cerebellum-dependent associative learning, measured by trace eyeblink conditioning (Zhang et al., 2019), in which CS and US are separated by a short pause, the trace interval. Reversible inactivation of mPFC through NMDA blockade during the acquisition and early consolidation phases of trace eyeblink conditioning slowed CR acquisition and slightly impaired CR expression in rats. In contrast, mPFC inactivation did not affect either acquisition or consolidation in the delay variation of the task (Takehara-Nishiuchi et al., 2005). Addressing functional connectivity between the mPFC and the cerebellum, Watson et al. found that electrical stimulation of the rat’s prelimbic subdivision of the mPFC (PL) evoked complex spikes in PCs within cerebellar lobule VII (Watson et al., 2009). This finding was complemented by observed connectivity from the caudal mPFC to the lateral pontine nuclei, which in turn sends the mossy fiber collaterals to the aIN, and is important for an adequate CR in trace eyeblink conditioning (Kalmbach et al., 2009). Consequently, it was then proposed that forebrain inputs to the cerebellum through the pontine nucleus may regulate the effect of the CS in the cerebellum, providing adequate timing between CS and US signal to instruct LTD (Weiss and Disterhoft, 2011). Thus, CS timing and duration are crucial for determining mPFC involvement in eyeblink conditioning. It was further demonstrated that the mPFC is also recruited when there is a low intensity US (Oswald et al., 2009) and CS (Wu et al., 2012, 2018; Li et al., 2018), therefore being necessary for optimal CR expression in suboptimal conditions. In addition, some studies have focused on how the mPFC fits into the brain network involved in defensive states. Pharmacological inactivation of the dorsal mPFC and CEA in well-trained mice severely attenuated the CR of trace eyeblink conditioning. Interestingly, tracings have shown that both regions project to different basilar pons regions, which overlap with projections toward the cerebellar cortex (Siegel et al., 2015). All these results combined suggest that the mPFC plays a role in regulating timing and intensity of CS and US in learned defensive responses dependent on the cerebellum, possibly acting together with other extracerebellar structures such as the amygdala (Lee and Kim, 2004; Wu et al., 2018).

With the goal to trace the connectivity between the cerebellum and the forebrain, a recent study combined injections of retrograde and anterograde adeno-associated viruses (AAV), and anterograde-transported herpes simplex virus (HSV) among all different divisions of the cerebellar cortex and their targets (Pisano et al., 2021). Strong projections to the anterior cingulate cortex were observed specifically stemming from lobules I–V, whereas lobules VI–X send denser projections to the infralimbic, PL, and orbital cortex (Pisano et al., 2021). Furthermore, disynaptic connections of the cerebellar cortex with the thalamus, synapsing within the DCN were revealed. Specifically, a moderate to strong connectivity from crus I and vermal lobules I–VII and crus II to ventromedial and ventral anterior lateral thalamic nuclei was found. Moreover, the ventral posteromedial nucleus receives input from all regions of the cerebellum, especially the posterior part. The thalamic reticular nucleus, the lateral posterior and mediodorsal nuclei, the lateral and medial geniculate nuclei and the zona incerta also receive input from the cerebellum. The thalamus in turn sends projections to the ipsilateral neocortex (layers 5, 6a, and 6b), the somatosensory, and the somatomotor cortex, with denser labeled neurons in the infralimbic, orbital, and PL areas, as previously reported (Pisano et al., 2021). In accordance with these tracing data, a previous study performed in rats has shown that electrical stimulation of the FN evoked field potentials in the PL (Watson et al., 2014), indicating a connectivity from the FN to the PL. Although several patterns of response were observed, most of the cells decreased their firing rates during FN stimulation, with a subsequent rebound increase. Interestingly, when cell activity within these two brain areas was simultaneously recorded during the open field assay, a significant network coherence was found during active locomotion, but not during rest, suggesting a cerebello-prefrontal pathway which is important during goal-directed behaviors. Together with the results from Watson et al., 2009, there may be a closed-loop circuit between the cerebellum and the PL, as reported in other areas of the PFC and the cerebellum (Kelly and Strick, 2003). However, it remains elusive whether this pathway is involved in defensive states. Taken together, the findings presented in this section demonstrate that threat memories involve the cerebellum as an important modulator characterized by precise timing of its neuronal contribution. This is in line with the idea of a cerebellar contribution to prediction errors generated to drive plasticity in mnemonic target circuits.

## 4. Cerebellar contribution to innate behavioral threat responses

In addition to the well-addressed cerebellar role in learned defensive behavioral responses, the cerebellum has been shown to play a role in innate defensive responses as well. Lesions within the rat cerebellar vermis decreased innate fear, seen by an increase in approach behavior in the cat test and enhancement of motor activity in the open field test in comparison with the controls (Supple et al., 1987). Additionally, in a recent study, a spinocerebellar ataxia mouse model with alterations exclusively to PCs was accessed in innate fear- and anxiety- related assays, such as the open field, elevated plus maze, and light/dark place preference assay (Bohne et al., 2022). Affected mice spent more time in the center and intermediate area of the open field, in the open arms of the elevated plus maze, and in the light zone of the light/dark box, thereby suggesting an anxiolytic-like phenotype of the spinocerebellar ataxia mouse model. However, when the threat level was escalated, i.e., when mice were exposed to aversive sounds, bright light conditions, or a looming stimulus, mice exhibited enhanced anxiety-like behavioral patterns. These aberrant defensive behavioral responses in mice with spinocerebellar ataxia (Bohne et al., 2022) suggest that cerebellar degeneration affects innate defensive responses in a complex manner, possibly *via* impacting correct assessment of threat levels. Curiously, patients with spinocerebellar ataxia showed reduced predictive and reactive motor timing tasks (Broersen et al., 2016), thereby confirming cerebellar involvement in timing function and generation of prediction errors and suggesting a putative similar mechanism for regulating innate defensive responses.

Recent studies have added a circuit-centered perspective on cerebellar contributions to innate defensive behavior. By combining electrophysiology and pharmacology, it was suggested that FN glutamatergic neurons regulate threat-related freezing behavior *via* projections to the vlPAG. More specifically, it was proposed that FN neuronal activity regulates intra-PAG dopaminergic modulation of a specific glutamatergic neuronal output population in the vlPAG (Vaaga et al., 2020). Thus, FN output impinges onto an already known defense-related pathway, in which inhibitory interneurons within the vlPAG control glutamatergic outputs to pre-motor neurons in the magnocellular nucleus of the medulla that drive conditioned and innate freezing behavior (Tovote et al., 2016). These Chx10- expressing vlPAG neurons have recently been demonstrated to mediate not only freezing behavior, but a short-lasting defensive microstate consisting of immobility concomitant with bradycardia (Signoret-Genest et al., 2023). The FN-PAG axis could therefore present a pathway through which the cerebellum contributes to the regulation of cardiac function during defensive states. Beyond regulation of defensiveness, the PAG constitutes a major output structure of the so called emotional motor system (Holstege). In fact, PAG circuits have been implicated in responses such as respiration, vocalization, coughing, sneezing, vomiting, micturition, defecation, parturition, ejaculation, and mating posture, motor functions intricately linked to not only aversive, but also rewarding emotions (Holstege, 1992, 2014). For instance, despite generating immobility in defensive states, the PAG also controls the immobility required for receptive female behavior (Van der Horst and Holstege, 1998). It is therefore conceivable that the cerebellum, through its modulatory connections to the PAG is involved in regulation of a broad range of emotional states.

## 5. Cerebellar regulation of cardiac defensive states

Early on, human cerebellar lesion studies provided evidence for a role of the cerebellum in higher cognitive functions and emotions. The cerebellar cognitive affective syndrome was first described in the late 90s and is characterized by impairments in executive function, visual-spatial memory, language production, and personality changes. This condition is observed in patients with lesions involving the posterior lobe of the cerebellum and the vermis (Schmahmann and Sherman, 1998). Furthermore, lesions involving mostly the FN have been associated with abnormal cardio-respiratory responses. For instance, a patient with vascular lesions in a part of the FN and the anterior and posterior IN showed pupil dilation, hyperventilation, bradycardia, and face flushing while attempting to move (Haines et al., 1997). This and other studies motivated investigations in animal models on cerebellar mechanisms underlying its role in regulation of autonomic emotional responses.

The first studies addressing a possible contribution of the cerebellum to cardiovascular dynamics were made in cats, where cerebellar ablation interfered with the distribution of cardiac output (Sheridan and Reis, 1972). Specifically, FN electrical stimulation resulted in an increase in heart rate and arterial pressure (Achari and Downman, 1970), constituting the so called fastigial pressor response (FPR). Electrophysiological studies performed in cats also showed that neurons within the rostral FN are responsive to changes in arterial blood pressure and respiratory stimuli (Lutherer et al., 1989), reinforcing the FN neurons‘ role in cardio-respiratory activity. Similarly, a later study performed in rats demonstrated a decrease in FN activity during phenylephrine-induced hypertension, while FN activity increased upon sodium nitroprusside-induced hypotension (Rector et al., 2006). Moreover, electrolytic lesions in the rat nucleus reticularis gigantocellularis, medial longitudinal fasciculus, rostral ventrolateral medulla, superior cerebellar peduncle, and NTS prevented the FPR (Giuditta et al., 2003), indicating a possible role of these structures in cardiac responses mediated by the FN. Furthermore, the paramedian reticular nucleus, which receives input from the FN as well as cardiovascular afferent input, has been shown to be related to the FPR as well (Miura and Reis, 1971; Elisevich and Ciriello, 1988). Indeed, previous studies have pointed out the connectivity between the FN and the NTS. The NTS is the major recipient of vagal afferents from the body, including ascending projections carrying interoceptive cardiac information. Injections of horseradish peroxidase within the intermediate region of the NTS, known for its cardiovascular control, resulted in dense retrograde labeling of the rostral and ventromedial FN in rats (Ross et al., 1981). In dogs, injections of [3H] leucine and [3H] proline at the same FN coordinates in which electrical stimulation promoted the FPR resulted in anterograde labeling throughout the caudal half of the NTS (Andrezik et al., 1984). Interestingly, the NTS also sends projections to the cerebellum. Injections of horseradish peroxidase within the anterior vermis of cats retrogradely labeled the caudal part of NTS, while posterior vermal injections resulted in retrograde labeling in the rostral part of the NTS (Somana and Walberg, 1979).

These results indicate an association of FN neuronal and autonomic activity and strongly suggest a cerebellar role in the regulation of autonomic function (Nisimaru, 2004). Importantly, these relatively unexplored regulatory mechanisms could involve both, descending control of cardiac function through DCN output as well as integration and modulation of ascending interoceptive cardiac information from the NTS.

In addition to a putative role in innate cardiac function, other studies addressed how the cerebellum may mediate learned autonomic responses. Specifically, the role of the vermis in cardiac responses was investigated using rats (Supple and Leaton, 1990a), which were first submitted to an aspirate lesion surgery throughout the vermis and subsequently underwent a threat conditioning paradigm. The heart rate was measured during exposure to a neutral tone in the habituation phase, and after pairing the tone-CS with a foot shock US during the fear conditioned bradycardia (conditioned heart rate). Vermal lesions attenuated the acquisition of CS-induced conditioned bradycardia without interfering with the resting heart rate and heart rate responses to a neutral tone and the US (Supple and Leaton, 1990a). However, bilateral lesions within the cerebellar cortex excluding the vermis and the paravermis did not affect heart rate conditioning in rats (Supple and Leaton, 1990b), therefore suggesting that the vermis is an essential area for the conditioned cardiac response. Accordingly, lesions within the IN and DN, downstream targets of the paravermis and lateral hemisphere, respectively, did not affect conditioned bradycardia in rabbits (Lavond et al., 1984). Further studies in rabbits addressed vermal topographic specificity of the conditioned cardiac response. Removal of the posterior vermis before heart rate conditioning significantly reduced the acquisition of conditioned bradycardia, without affecting the innate or the resting heart rate. However, when the posterior vermis was removed after the acquisition of the CR, no effect was observed (Sebastiani et al., 1992). These findings indicate that the posterior vermis is important for the acquisition, but not the retention of conditioned cardiac defensive responses. Conversely, lesions within the anterior vermis of well-conditioned rabbits eliminated prior conditioned bradycardia, without affecting innate cardiac responses (Supple and Kapp, 1993). Likewise, PCs of rabbits’ anterior vermis increased their firing in response to the CS-US pairing in heart rate conditioning. Moreover, a correlation between the magnitude of these CS-responsive PCs and conditioned bradycardia intensity was seen (Supple et al., 1993). Taken together, these early results established the anterior vermis as a critical site for the acquisition and retention of learned cardiac defensive responses. A more recent study performed in mice investigated the part of an important cerebellar input source, the inferior olive, in cardiac defensive responses (Kotajima et al., 2014). Mice with inferior olive lesions exhibited an impaired acquisition and expression of conditioned bradycardia, as well as an attenuation of US-induced tachycardia. However, inferior olive lesions did not interfere with the resting heart rate or with the heart rate responses to a neutral tone (Kotajima et al., 2014). This data not only implies that the inferior olive conveys the US information to the cerebellum (Mauk et al., 1986) during heart rate conditioning, but also suggests a possible role of the inferior olive in learned cardiac defensive responses. However, it is yet to be determined how the inferior olive interacts with the anterior vermis and the FN to generate appropriate heart rate responses in defensive states.

## 6. The cerebellum as an integrator of defensive state components

Defensive states are multimodal, highly dynamic processes that crucially depend on brain-body interactions, including behavioral, cardio-respiratory, and endocrine responses that enable the expression of proper responses in reaction to threat. These complex, integrated defensive responses were recently studied by Signoret-Genest et al. who developed a novel framework for combined analysis of defensive behaviors and their associated cardiac responses, i.e., heart rate changes in different anxiety and fear-related assays (Signoret-Genest et al., 2023). This study emphasizes the integrated nature of defensive states and puts forth the concept of short-lasting microstates and long- lasting macrostates, which interact with each other to generate complex defensive state dynamics. This means, for example, that identical threat-related behaviors can be associated with different cardiac outputs, depending on the pre-existing state of the animal. Importantly, the dynamic heart rate responses are a highly sensitive indicator of integrated defensive microstates, which include behavioral and cardiac compounds, rather than just a mere result of behavioral responses (Signoret-Genest et al., 2023).

Although past researchers have tried to combine behavioral and cardiac outcomes to evaluate the cerebellar role in defensive states (Lavond et al., 1984), most recent studies focus on the behavioral component, thereby missing important parts of complex defensive responses needed for a comprehensive understanding of integrated defensive states. When it comes to regulation of behavioral defense responses, a role for the cerebellum has firmly been established. Taking into account the integrated nature of behavioral motor and cardiac visceromotor responses, the question arises whether and how the cerebellum influences the cardiac defense components as well, and if so, through which mechanism. Recently, cerebellar outputs have been demonstrated to affect vlPAG neuronal activity, and specifically modulating the activity of Chx10-positive neurons. Because of the suggested role of these neurons as freezing-bradycardia microstate generator elements (Signoret-Genest et al., 2023), it is conceivable that the cerebellum plays a role in defensive state regulation through the FN-vlPAG pathway. The precise underlying circuit mechanisms putatively involving functionally distinct FN modules (Fujita et al., 2020) need to be investigated in the future. Whereas there is evidence for further functional specificity of FN-vlPAG pathways in that other FN glutamatergic inputs to the PAG subserve learned, but not innate defensive behaviors, FN projections to the parafascicular thalamic nucleus were shown to modulate defensive behaviors in classical rodent assays for anxiety (Frontera et al., 2020). These behavioral patterns are associated with specific cardiac dynamics, thereby constituting a defensive state space defined by combinations of multimodal, adaptive threat responses (Signoret-Genest et al., 2023).

The precise mechanisms underlying integration of these multidimensional and highly dynamic states are largely unexplored. Given the cerebellar involvement in cardiac responses, i.e., innate and learned heart rate changes, and its regulatory role in innate and learned defensive behavioral responses, is reasonable to hypothesize that the cerebellum contributes to integration of different defensive states components. As previously discussed, the cerebellum processes sensory information to adjust ongoing behavior in a moment-to-moment manner and to provide teaching signals for threat learning. As a result, the system adapts expression of defensive state dynamics to the current and anticipated threat level. Defensive state dynamics are not only determined by exteroceptive sensory inputs, but also crucially by interoceptive feedback from the body (Hsueh et al., 2023), two information streams that have to be integrated into an internal model to predict and adequately respond to threats (Barrett, 2017; Kleckner et al., 2017). Based on the evidence presented in this review, it is likely that both, intracerebellar circuits as well as cerebellar connections to classic defensive circuits crucially contribute to these integrative and regulatory processes.

From an evolutionary perspective, the importance of fine-tuning of defensive responses likely increased together with the expansion and enhanced complexity of the defensive state repertoire from rodents to primates. It could be speculated that therefore, that the cerebellar role in predicting and regulating integrated defensive states might more prominent in humans, as corroborated by severe symptoms of the cognitive-affective syndrome associated with cerebellar lesions in children. In line, it is also noteworthy that the cerebellum appears to have expanded throughout evolution of primates even more than neocortex, thus reflecting in general, a more important role in fine-tuning of motor and non-motor functions (Barton and Venditti, 2014). Nonetheless, in lower vertebrates and especially in prey animals such as mice, adequate moment-to-moment defensive state regulation is particularly important to ensure survival, suggesting an important role of predictive and integrative threat-related cerebellar mechanisms. However, because large aspects of responses to threat have been conserved, the investigation and modeling of the cerebellum’s role in defensive states in rodents is a promising avenue toward a better understanding of human cerebellar function. Future circuit-centered studies combining observational and pertubation approaches are required to elucidate these cerebellar contributions, such as putative prediction error mechanisms. The challenges will be to gather temporally highly resolved data from identified circuit elements, that is, individual neurons, while at the same time integrate the network perspective by synchronous recordings and subsequent, pinpointed manipulation of entire neuronal ensembles.

## Author contributions

All authors listed have made a substantial, direct, and intellectual contribution to the work, and approved it for publication.
